# Expression quantitative trait loci in the developing human brain and their enrichment in neuropsychiatric disorders

**DOI:** 10.1186/s13059-018-1567-1

**Published:** 2018-11-12

**Authors:** Heath E. O’Brien, Eilis Hannon, Matthew J. Hill, Carolina C. Toste, Matthew J. Robertson, Joanne E. Morgan, Gemma McLaughlin, Cathryn M. Lewis, Leonard C. Schalkwyk, Lynsey S. Hall, Antonio F. Pardiñas, Michael J. Owen, Michael C. O’Donovan, Jonathan Mill, Nicholas J. Bray

**Affiliations:** 1 MRC Centre for Neuropsychiatric Genetics & Genomics, Division of Psychological Medicine & Clinical Neurosciences, Cardiff University, Cardiff, UK; 2 University of Exeter Medical School, University of Exeter, Exeter, UK; 3 Institute of Psychiatry, Psychology & Neuroscience, King’s College London, London, UK; 4 School of Biological Sciences, University of Essex, Colchester, UK; 5 MRC Centre for Neuropsychiatric Genetics & Genomics, Cardiff University School of Medicine, Hadyn Ellis Building, Maindy Road, Cardiff, CF24 4HQ UK

## Abstract

**Background:**

Genetic influences on gene expression in the human fetal brain plausibly impact upon a variety of postnatal brain-related traits, including susceptibility to neuropsychiatric disorders. However, to date, there have been no studies that have mapped genome-wide expression quantitative trait loci (eQTL) specifically in the human prenatal brain.

**Results:**

We performed deep RNA sequencing and genome-wide genotyping on a unique collection of 120 human brains from the second trimester of gestation to provide the first eQTL dataset derived exclusively from the human fetal brain. We identify high confidence *cis*-acting eQTL at the individual transcript as well as whole gene level, including many mapping to a common inversion polymorphism on chromosome 17q21. Fetal brain eQTL are enriched among risk variants for postnatal conditions including attention deficit hyperactivity disorder, schizophrenia, and bipolar disorder. We further identify changes in gene expression within the prenatal brain that potentially mediate risk for neuropsychiatric traits, including increased expression of *C4A* in association with genetic risk for schizophrenia, increased expression of *LRRC57* in association with genetic risk for bipolar disorder, and altered expression of multiple genes within the chromosome 17q21 inversion in association with variants influencing the personality trait of neuroticism.

**Conclusions:**

We have mapped eQTL operating in the human fetal brain, providing evidence that these confer risk to certain neuropsychiatric disorders, and identifying gene expression changes that potentially mediate susceptibility to these conditions.

**Electronic supplementary material:**

The online version of this article (10.1186/s13059-018-1567-1) contains supplementary material, which is available to authorized users.

## Background

Results from genome-wide association studies (GWAS) have established an important role for common non-coding genetic variation in complex diseases [1, 2]. These associations are likely to reflect variants that influence gene expression, through effects on RNA transcription, splicing, or stability. By combining genome-wide genotyping with transcriptome profiling, variants associated with altered gene expression can be mapped on a genomic scale as expression quantitative trait loci (eQTL) [3, 4]. Although eQTL are often shared between tissues, they can also operate in a tissue-specific manner [4–6], as a result of cellular differences in the expression of the *trans-*regulators that interact with these loci [7], as well as in the epigenetic modification and accessibility of the regulatory elements in which they are located [8, 9].

Genetic influences on gene regulation that are active during human prenatal brain development plausibly impact upon a variety of postnatal, brain-related phenotypes. For example, we have previously shown that genetic variants associated with DNA methylation in the human fetal brain are enriched among variants associated with schizophrenia [10], a severe psychiatric disorder with a hypothesized early neurodevelopmental component [11, 12]. Common genetic variants associated with schizophrenia are also reported to be enriched within open chromatin (an index of regulatory regions) in the developing human cerebral cortex [13], and there are reports of association between gene expression in fetal brain and schizophrenia risk alleles at individual loci, e.g., [6, 14]. However, apart from one previous investigation that included a relatively small subset of fetal brains (*n* = 38) [15], no study has explored genetic effects on gene expression in the human prenatal brain on a genome-wide scale.

In the present study, we carried out deep RNA sequencing and genome-wide genotyping on a large collection of human brains from the second trimester of gestation (*n* = 120), providing the first eQTL dataset derived exclusively from the prenatal human brain. We identify high confidence *cis*-acting eQTL at the individual transcript as well as whole gene level, including many mapping to a common inversion polymorphism on chromosome 17q21. We further show that fetal brain eQTL are enriched among risk variants for neuropsychiatric disorders and identify genetically influenced changes in gene expression within the developing brain that potentially mediate risk for these conditions.

## Results

We performed strand-specific, whole transcriptome sequencing of total RNA derived from brain tissue from 120 human fetuses aged 12–19 post-conception weeks (PCW), generating a median 119 million read pairs per sample (Additional file 1: Table S1). Expression measures were derived for 144,448 Ensembl transcripts, annotated to 28,875 genes. Genomic DNA from each sample was genotyped for approximately 710,000 single nucleotide polymorphisms (SNPs), followed by genotype imputation using the Haplotype Reference Consortium r1.1 panel [16]. After strict quality control, 5.8 million SNPs were retained for analysis. Consistent with the GTEx Consortium [4], we controlled gene expression measures for latent factors using probabilistic estimation of expression residuals (PEER) [17] in addition to specified covariates and principal components from the genotyping matrix (Additional file 2: Figure S1 and Figure S2). *Cis*-eQTL were identified by linear regression of allele dosage against adjusted, quantile-normalized gene expression measures using FastQTL [18].

### Characterization of *cis*-eQTL in the human fetal brain

**Fig. 1:**
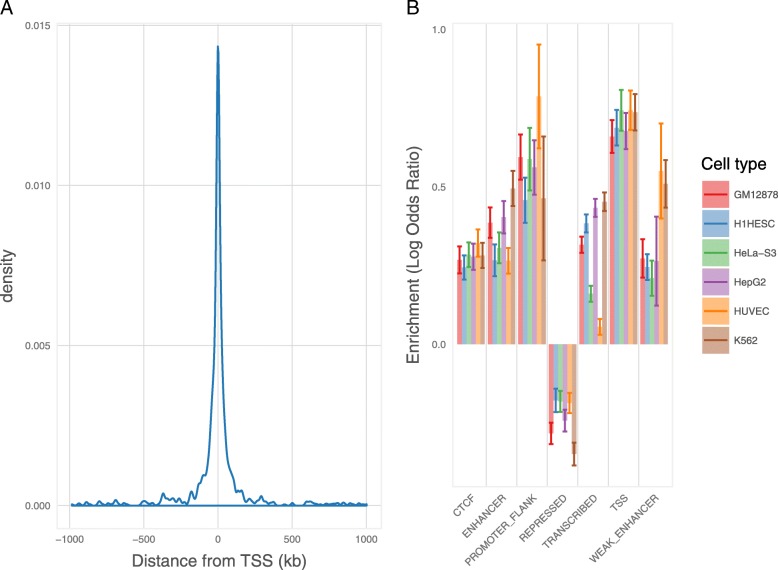
Genomic characterization of fetal brain *cis*-eQTL. **a** Plot of eQTL density (FDR < 0.05) versus distance from transcription start site for all eTranscripts. Expression QTLs mapping to a common 900-kb inversion on chromosome 17q21 were excluded from this analysis due to extensive linkage disequilibrium across the inversion. **b** Enrichment of eTranscript eQTL within genomic features identified by the ENCODE [8] and Roadmap Epigenomics Consortium [9] projects in six human cell lines. Enrichments are expressed as the natural log of odds ratios, with error bars representing 95% confidence intervals. GM12878 = lymphoblastoid cell line; H1HESC = embryonic stem cell line; HeLa-S3 = cervix adenocarcinoma cell line; HepG2 liver carcinoma cell line; HUVEC = umbilical vein endothelial cells; K562 = myeloid leukemia cell line. Fetal brain eQTL are significantly enriched in regions annotated as TSS, flanking promoter, enhancer, weak enhancer, and CTCF binding sites, but significantly depleted in repressed genomic regions

We identified *cis*-eQTL (defined as variants within 1 Mb of the transcription start site [TSS] of the regulated gene) associated with the fetal brain expression of 1329 genes (eGenes) and 3252 individual transcripts (eTranscripts) at a false discovery rate (FDR) < 0.05 (Additional file 1: Table S2 and Table S3). The majority (86%) of eQTL detected at the whole gene level were also detected at the individual transcript level. Consistent with previous eQTL mapping studies [5, 15, 19], significant eQTL were concentrated in proximity to the TSS of the regulated transcript (Fig. 1a). We further tested for enrichment of high confidence *cis-*eQTLs (FDR < 0.05) within functional annotations provided by the ENCODE [8] and Roadmap Epigenomics Consortium [9] projects (Additional file 1: Table S4 and Table S5). Fetal brain *cis-*eQTL were significantly enriched within regions annotated as TSS, flanking promoters, enhancers, weak enhancers, and CTCF binding sites, but significantly depleted in repressed regions (Fig. 1b). Identified *cis*-eQTL were also notably enriched within regions containing histone modifications indicative of regulatory activity (Additional file 2: Figure S3). We additionally tested for enrichment, among our high confidence *cis*-eQTL, of variants that we have previously found to be associated with altered DNA methylation (mQTL) in the human fetal brain [10]. Consistent with an association between DNA methylation and gene expression [20, 21], fetal brain mQTL were enriched sixfold among fetal brain *cis*-eQTL (*P* = 3.13 × 10^− 19^).

### eQTL at a common inversion polymorphism on chromosome 17q21

Twenty-one of the *cis*-eQTL eGenes identified in fetal brain are located within a region of chromosome 17q21.31 containing a common 900-kb inversion polymorphism and several common duplications [22, 23]. Variants within this inversion (denoted “H2”), which appears to be under positive selection in Europeans [22], have recently been implicated in neuroticism [24–26], while markers on the non-inverted form (denoted “H1”) are associated with several neurodegenerative diseases [27–29]. For 13 of the eGenes in this region (e.g., *KANSL1*, *LRRC37A*, *DND1P1*), *cis-*eQTL could be largely explained by inversion status (r2 with H1/H2 tag SNP rs1800547 > 0.9), with variants tagging the inverted H2 form associated with both increases and decreases in gene expression (Table 1). For other genes in the region (e.g., *NSF*, *ARL17A*, *ARL17B*), identified *cis*-eQTL might index duplicated expressed gene sequence as well as regulatory variants that have arisen independently on the H1 or H2 haplotypes.

**Table 1:** Fetal brain eGenes located at a common inversion polymorphism on chromosome 17q21

eGene	ENSEMBL ID	Number eQTL SNPs FDR < 0.05	Top eQTL	Top eQTL FDR	*r*^2^ with inversion*	H1/H2 expression ratio
*NSF*	ENSG00000073969	40	rs3874943	4.05E−06	0.14	1.02
*KANSL1*	ENSG00000120071	2226	rs2668690	2.81E−14	0.94	0.89
*LRRC37A*	ENSG00000176681	2225	rs3785884	5.89E−17	0.97	0.80
*ARL17A*	ENSG00000185829	34	rs1863115	7.03E−04	0.11	1.00
*KANSL1-AS1*	ENSG00000214401	2215	rs62057151	1.72E−11	0.94	0.81
*LRRC37A4P*	ENSG00000214425	2210	rs55944735	7.40E−12	0.97	1.96
*ARL17B*	ENSG00000228696	48	rs1863115	1.82E−06	0.11	1.02
*LRRC37A2*	ENSG00000238083	2213	rs753236	7.11E−15	0.94	0.83
*NSFP1*	ENSG00000260075	54	rs1863115	2.89E−14	0.11	1.22
*AC005670.2*	ENSG00000262633	1036	rs2696689	4.02E−03	0.65	0.91
*MAPK8IP1P2*	ENSG00000263503	2276	rs111447859	1.50E−23	0.97	0.39
*DND1P1*	ENSG00000264070	2199	17:44219831	1.73E−08	0.97	1.38
*MAPT-AS1*	ENSG00000264589	1816	rs60814418	3.01E−02	0.61	1.15
*RN7SL199P*	ENSG00000265315	2203	rs56180212	2.47E−09	0.97	0.78
*RN7SL656P*	ENSG00000265411	2204	rs56180212	2.17E−09	0.97	0.78
*AC138645.1*	ENSG00000266497	2218	rs4327090	5.75E−11	0.94	0.82
*AC091132.3*	ENSG00000266504	2213	rs4327090	5.12E−11	0.94	0.82
*AC091132.4*	ENSG00000266918	2066	rs9899833	1.55E−02	0.54	1.06
*AC091132.5*	ENSG00000267198	39	rs79475968	7.94E−05	0.04	1.03
*AC091132.6*	ENSG00000267246	2253	rs753236	7.61E−11	0.94	0.76
*Metazoa_SRP*	ENSG00000278770	2215	rs4327090	2.30E−09	0.94	1.54

*Consistent with Stefansson et al [22], inversion status was inferred through genotype at SNP rs1800547, with the *MAPT* haplotype “H2” used to denote the inversion and “H1” the non-inverted form

#### Tissue-sharing and evidence for fetal-specific eQTL

**Fig. 2:**
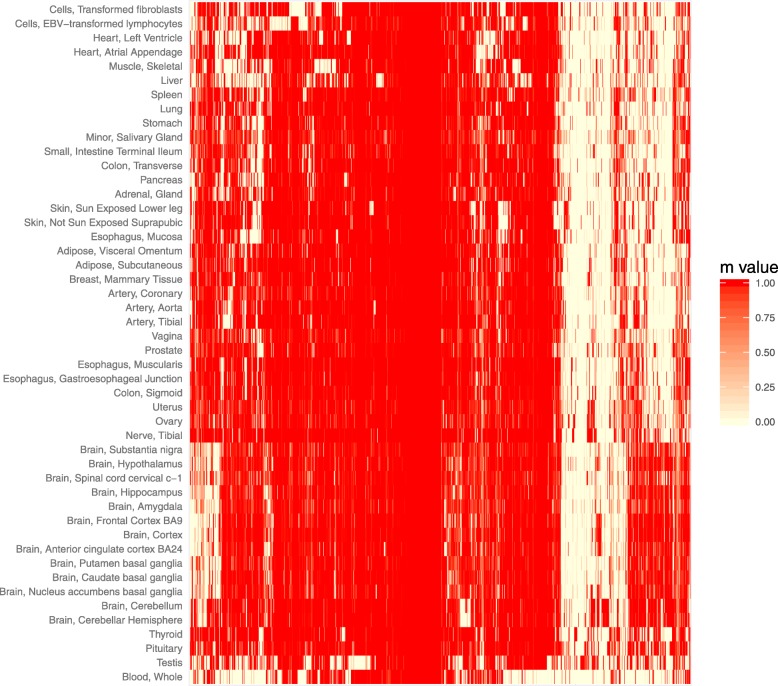
Heatmap of *m* values for fetal brain *cis*-eQTL (FDR < 0.05) for 974 eGenes across 48 adult tissues assayed by the GTEx Consortium [4]. *m* values are derived from a Meta-Tissue [68] analysis performed by the GTEx consortium (using v7 data), and estimate the posterior probability that an eQTL is shared between each tissue. GTEx *m* values are available for 974 gene fetal brain *cis*-eQTL that are predicted to be shared by at least two adult tissues (*m* values > 0.9); the five fetal brain *cis*-eQTL that are eQTL in only one adult tissue (FDR < 0.05) are not included. Greatest sharing of fetal brain eQTL is observed for adult brain regions, where, on average, 79% of the tested fetal brain eQTL are predicted to be shared

We next explored the extent to which *cis*-eQTL identified in the fetal brain are shared among 48 adult human tissues analyzed through the Genotype-Tissue Expression (GTEx) project [4]. For this, we restricted our analyses to the eQTL we identified for fetal eGenes as the GTEx project does not currently provide eQTL data at the individual transcript level. Of the 1151 fetal brain eGenes for which GTEx Consortium data were available, 979 had a top eQTL (the most significant fetal brain eQTL for that gene that was genotyped in the GTEx study) that was also observed in at least one adult tissue (at FDR < 0.05). The shared eQTL for 974 of these fetal eGenes were predicted to be active in at least two adult tissues. Tissue-sharing of fetal brain eQTL was most pronounced within regions of the adult brain, where an average of 79% of the tested fetal brain eQTL were predicted to be shared, and lowest for whole blood, where only 41% of the tested fetal brain eQTL were predicted to be shared (Fig. 2). Predicted tissue sharing was supported by estimates of π_1_ (the proportion of true positives [30]) among fetal eQTL when tested for replication in individual GTEx tissues, with high π_1_ estimates in adult brain regions despite relatively small GTEx sample sizes (Additional file 2: Figure S4). For 172 eGenes assayed by the GTEx Consortium, the eQTL identified in fetal brain were not associated with significant (FDR < 0.05) effects on their expression in any sampled adult tissue, and these might therefore constitute fetal-specific eQTL (Additional file 1: Table S6).

#### Regional and developmental expression of fetal eGenes in the human brain

We used RNA sequencing data from dissected regions of the human pre- and post-natal brain, available through the BrainSpan project, to explore the spatiotemporal expression of the fetal eGenes we identified. In general, fetal eGenes were expressed fairly uniformly across dissected regions of both the fetal and adult brain, with some genes showing very high expression and others very low (Additional file 2: Figure S5 and Figure S6). Similarly, the majority of fetal eGenes displayed a reasonably stable pattern of expression across brain development, even when the associated eQTLs were predicted to be fetal-specific (Additional file 2: Figure S7). A notable exception to this pattern was observed in the developmental expression of the predicted fetal-specific eGene *HBG1*, encoding *hemoglobin subunit gamma 1* (a component of fetal hemoglobin), which showed the expected preponderance in prenatal brain (Additional file 2: Figure S8).

#### Enrichment of fetal brain *cis*-eQTL within genetic variants associated with neuropsychiatric traits

**Fig. 3:**
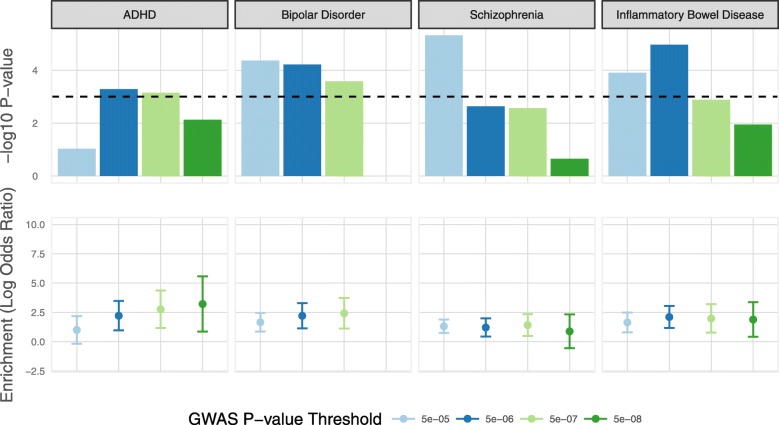
Enrichment of fetal brain transcript eQTL within variants associated with complex post-natal traits. Enrichments were tested at four GWAS *P* value thresholds (*P* < 5 × 10^− 5^, 5 × 10^− 6^, 5 × 10^− 7^, and 5 × 10^− 8^) for each trait. Top panel shows log10 *P* values at each threshold. The bottom panel shows the natural log of the odds ratio, with 95% confidence intervals, at each threshold. Only traits for which the significance of eQTL enrichment survives correction for multiple tests (*P* < 0.001; dotted line in upper panel) at one or more GWAS *P* value threshold are shown. There are no overlaps between fetal brain eQTL and risk variants for bipolar disorder at the *P* < 5 × 10^− 8^ threshold

Genetic effects on gene expression operating in the human fetal brain could influence a variety of post-natal, brain-related phenotypes. We tested for enrichment among high confidence fetal brain eQTL (FDR < 0.05) of variants associated with seven neuropsychiatric traits (attention deficit hyperactivity disorder, anorexia nervosa, autism spectrum disorder, bipolar disorder, major depressive disorder, neuroticism, and schizophrenia) using large-scale GWAS data [26, 31–36]. For these analyses, we focused on transcript-level eQTL as these largely encompass those identified at the gene level and are likely to additionally index subtle regulatory variation (e.g., those affecting splicing, or transcript-specific promoters/enhancers) that could be relevant to complex traits [37]. We assessed enrichment among fetal eQTL of trait-associated variants at four GWAS *P* value thresholds for each trait (*P* < 5 × 10^− 5^, 5 × 10^− 6^, 5 × 10^− 7^, and 5 × 10^− 8^), using a method that accounts for allele frequency, linkage disequilibrium, and local gene density [38]. As a comparison, we performed identical analyses on similar sized GWAS data for five non-brain traits (body mass index, coronary artery disease, inflammatory bowel disease, height, and type 2 diabetes) [39–43]. Fetal brain transcript eQTL were notably enriched (*P* < 0.001) within trait-associated variants for three neuropsychiatric disorders (attention deficit hyperactivity disorder, bipolar disorder, and schizophrenia) and one non-brain trait (inflammatory bowel disease) at one or more GWAS *P* value threshold (Fig. 3; Additional file 1: Table S7).

#### Identification of gene expression changes in fetal brain associated with neuropsychiatric traits

Given evidence for a potential role of fetal brain eQTL in neuropsychiatric traits, we next applied summary data-based Mendelian randomization (SMR) to identify genetically influenced changes in gene expression in the fetal brain that could mediate genetic risk for these conditions. This approach employs Mendelian randomization to test for pleiotropic associations between gene expression and complex traits using eQTL and trait GWAS summary data [44, 45]. We tested for joint associations between gene expression and risk for the seven neuropsychiatric traits (attention deficit hyperactivity disorder, anorexia nervosa, autism spectrum disorder, bipolar disorder, major depressive disorder, neuroticism, and schizophrenia) using the same GWAS datasets as we used for enrichment analyses [26, 31–36]. We considered only eGenes and eTranscripts for which fetal brain eQTL were identified at FDR < 0.05, and report associations that survive correction for multiple tests (eGenes, *P*-SMR = 3.76 × 10^− 5^; eTranscripts, *P*-SMR < 1.54 × 10^− 5^). In addition, we only report associations that are non-significant (*P* > 0.05) for the HEIDI (heterogeneity in dependent instruments) test, indicating that they are unlikely to be driven by linkage between the eQTL and independent risk variants [44]. In total, we identified 15 eGenes and 34 eTranscripts where expression in fetal brain was pleiotropically associated with at least one neuropsychiatric trait and conformed to the above criteria (Additional file 1: Table S8 and Table S9, respectively).

**Fig. 4:**
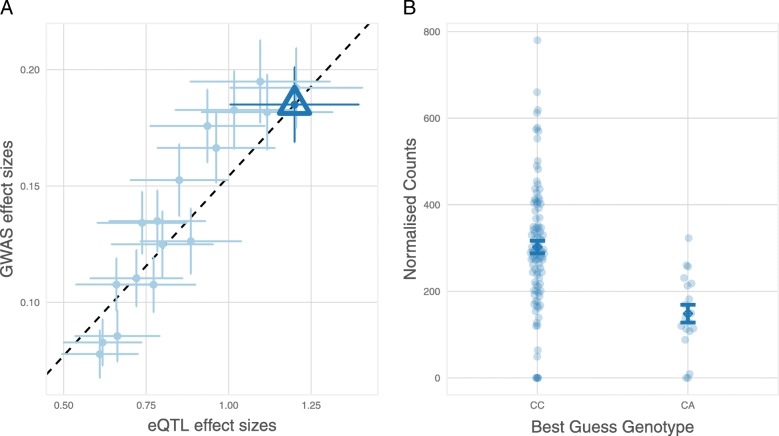
Association between expression of the Complement C4A gene in fetal brain and genetic risk for schizophrenia. **a** Effect sizes of SNPs from schizophrenia GWAS [36] plotted against their effect sizes as fetal brain *cis*-eQTL for *C4A*. The triangular data point indicates the variant with the lowest *P*-SMR. Error bars are the standard errors of SNP effects. **b** Homozygotes for the schizophrenia-associated C-allele of rs9267544 exhibit increased expression of *C4A* in fetal brain compared with heterozygotes for this SNP. Error bars represent standard errors

We identified 6 eGenes and 14 eTranscripts for which fetal brain expression was associated with schizophrenia risk. These include reduced expression of *ABCB9*, encoding *ATP Binding Cassette Subfamily B Member 9* (*P*-SMR = 2.11 × 10^− 5^), which has been previously implicated through SMR studies of schizophrenia GWAS in blood [44, 46] and other non-brain adult tissues [45], and increased expression of *CSPG4P11*, encoding *Chondroitin Sulfate Proteoglycan 4 Pseudogene 11* (*P*-SMR = 6.66 × 10^− 6^), which has also been reported in an SMR study of adult human brain gene expression data [45]. We also observed association between schizophrenia and reduced fetal brain expression of a transcript of *KLC1*, encoding *Kinesin Light Chain 1* (*P*-SMR = 2.95 × 10^− 7^), which has recently been implicated in schizophrenia risk through a splicing QTL in the adult brain [47]. The most significant association with schizophrenia (*P*-SMR = 6.3 × 10^− 8^) was in the expression of *C4A*, encoding *Complement C4A*, which exhibited increased expression in association with risk variation (Fig. 4). Genetic association between schizophrenia and the major histocompatibility complex (MHC) locus on chromosome 6 has been shown to partly reflect common gene copy number variants resulting in increased expression of *C4A* in the adult human brain [48]. The present data suggest that pathogenic effects of variation at the *C4* locus might begin in utero. We also observed several novel associations with schizophrenia, including reduced expression of transcripts of *FLOT1*, encoding *Flotillin 1* (*P*-SMR = 5.0 × 10^− 7^), increased expression of a transcript of *MSANTD2,* encoding *Myb/SANT DNA Binding Domain Containing 2* (*P*-SMR = 2.8 × 10^− 6^), and increased expression of *HIST1H4L*, encoding *Histone Cluster 1 H4 Family Member L* (*P*-SMR = 4.4 × 10^− 7^).

We observed association between variants influencing the personality trait of neuroticism and the expression of 7 eGenes and 18 eTranscripts in fetal brain. The majority of these (4 eGenes and 17 eTranscripts) were located at the common inversion polymorphism on chromosome 17q21. The most significant SMR associations were observed for transcripts of *LRRC37A*, encoding *Leucine Rich Repeat Containing 37A* (smallest *P*-SMR = 2.33 × 10^− 13^) and *KANSL1*, encoding *KAT8 Regulatory NSL Complex Subunit 1* (smallest *P*-SMR = 8.1 × 10^− 12^), with variants conferring higher neuroticism scores associated with increased expression of both genes. The three neuroticism-associated genes that did not map to the inversion are functionally uncharacterized, but for one of these genes (*AC022784.7*), the *cis-*eQTL is predicted to be fetal-specific. The only neuroticism-associated transcript that did not map to chromosome 17q21 is annotated to the *ORC4* gene, encoding *Origin Recognition Complex Subunit 4* (*P*-SMR = 1.91 × 10^− 7^).

Reduced expression of one transcript, annotated to the *RPL31P12* gene (encoding *Ribosomal Protein L31 Pseudogene 12*), was associated with major depressive disorder (*P*-SMR = 2.19 × 10^− 6^), while increased expression of the *RPS26* gene (encoding *Ribosomal Protein S26*) was associated with genetic risk for anorexia nervosa (*P*-SMR = 2.34 × 10^− 6^). Increased expression of the *LRRC57* gene, encoding *Leucine Rich Repeat Containing 57*, was associated with bipolar disorder (*P*-SMR = 3.07 × 10^− 5^). In addition, a transcript annotated to *GNL3*, encoding *G Protein Nucleolar 3*, was associated with both bipolar disorder (*P*-SMR = 2.17 × 10^− 6^) and schizophrenia (*P*-SMR = 2.71 × 10^− 7^), with risk alleles for both disorders associated with increased expression of this transcript in fetal brain.

We additionally explored whether eQTLs identified in the adult human brain (dorsolateral prefrontal cortex) [49] that have been implicated in neuropsychiatric traits in a previous SMR study [45] are potentially also operating in the fetal brain. For four fetal eGenes, we observed strong linkage disequilibrium in our samples (*r*^2^ > 0.8) between the most significant fetal brain eQTL and the adult brain eQTL implicated in schizophrenia risk through SMR (Additional file 1: Table S10). In addition to *CSPG4P11*, which showed a significant SMR association with schizophrenia in the present study, these included *CD46*, encoding the *CD46* complement regulatory protein, *GOLGA2P7*, encoding *GOLGA2 pseudogene 2*, and *SLCO4C1*, encoding S*olute Carrier Organic Anion Transporter Family Member 4C1*. There was no apparent overlap between adult brain eQTL implicated in neuroticism and eQTL regulating the fetal eGenes identified in this study.

## Discussion

We present the first genome-wide eQTL mapping study performed exclusively on the prenatal human brain. Through deep RNA sequencing, we have been able to interrogate eQTL at the individual transcript as well as whole gene level, providing a more precise elucidation of common genetic influences on gene expression in the developing brain and the molecular differences that could confer susceptibility to neuropsychiatric disorders.

Using a method that controls for the number of SNPs tested at each locus as well as the number of genes/transcripts assayed [4, 18], we identified high confidence *cis-*eQTL regulating 1329 genes and 3252 individual transcripts in the human fetal brain. This approximates the number of eGenes identified using the same procedure in adult brain collections of comparable size; for example, in 92 samples from the adult frontal cortex, the GTEx Consortium identified *cis*-eQTL (FDR < 0.05) for 1588 genes [4]. However, this is likely to constitute only a small percentage of the genes expressed in fetal brain that are subject to variable genetic *cis-*regulation. Studies in adult human tissues have indicated that a sizeable proportion of expressed genes are variably *cis*-regulated [4, 50, 51], with eQTL mapping studies showing the expected strong correlation between eQTL discovery and sample size [4]. Larger collections of fetal brain are therefore likely to yield many additional eQTL, with resulting improvements in power to identify gene expression changes relevant to brain-related traits.

Many of the fetal brain eQTL we identified map to a common 900-kb inversion polymorphism on chromosome 17q21.31. The eQTL for 13 of the genes in the region are largely tagged by the inversion, which was associated with both increases and decreases in gene expression. Although our analyses of tissue-sharing suggest that none of the eQTL mapping to this region are specific to the fetal brain, only one study to our knowledge has specifically reported any of the gene expression differences we observe [52]. This previous study reported differences between H1 and H2 in the expression of six genes in the adult human brain, which, congruent with our data, included increased expression of *LRRC37A* in association with the inverted H2 form.

Of the tested neuropsychiatric traits, we observed notable enrichment among high confidence fetal brain eQTL of variants associated with attention deficit hyperactivity disorder (ADHD), bipolar disorder, and schizophrenia. We also observed nominally significant enrichment of variants associated with autism at lower GWAS *P* value thresholds (Additional file 1: Table S7), although we note that the number of tested variants for this was smaller than for other brain-related traits, limiting statistical power. Like autism, ADHD is considered to be a neurodevelopmental condition, but the genes involved and the developmental timing of their action are largely unknown. We observed an average 13-fold enrichment of ADHD-associated variants within fetal brain eQTL across the four GWAS *P* value thresholds tested, suggesting an important prenatal genetic component to this condition. Our finding of an enrichment of schizophrenia-associated variants among fetal brain eQTL is also consistent with the long hypothesized early neurodevelopmental component to this disorder [11, 12]. Although less commonly conceptualized as neurodevelopmental in origin, we observed a similar enrichment of risk variants for bipolar disorder among high confidence fetal brain eQTL. In contrast, we observed no enrichment of risk variants for major depressive disorder, despite similar numbers of tested risk-associated variants at each GWAS *P* value threshold (Additional file 1: Table S7). Among non-brain traits, the most pronounced enrichment of fetal brain eQTL was observed for variants associated with inflammatory bowel disease (average sevenfold enrichment across the four GWAS *P* value thresholds), suggesting a developmental component to this condition.

The eQTL giving rise to the identified pleiotropic associations between gene expression in the fetal brain and risk for neuropsychiatric disorders do not, for the most part, appear to be fetal-specific. However, their activity within the brain at this timepoint makes it plausible that their pathogenic effects start prenatally, even if they continue into adulthood. Indeed, several of the genes that we implicate are known to have important roles in early brain development. For example, leucine-rich repeat containing genes (which include *LRRC37A*, implicated in neuroticism, and *LRRC57*, implicated in bipolar disorder) are known to be key organizers of excitatory and inhibitory synapse formation [53]. Similarly, *FLOT1*, which exhibited reduced expression in association with genetic risk for schizophrenia, has been found to promote the formation of hippocampal glutamatergic synapses [54], which appear to be decreased in the disorder [55]. Increased expression of *C4A* in association with schizophrenia risk variation was originally reported in the adult brain [48], where the authors provided data to support a role for this gene in synaptic pruning. Although this process is important for maturation of regions such as the frontal cortex in adolescence, it begins (and is for some brain regions pronounced) during the perinatal period [56]. Our findings thus suggest that as well as influencing synaptic pruning during adolescence, effects of *C4* risk variation could initially impact neural connectivity at this early phase of synaptic refinement.

## Conclusion

We have mapped high confidence eQTL operating in the human fetal brain. Although a large proportion of these continue to operate in adulthood, their activity at this timepoint and enrichment among risk variants for certain neuropsychiatric disorders suggest that their influence on brain-related traits may at least start at this early stage of development. We identify several genes and individual transcripts for which expression in the fetal brain could mediate genetic risk for neuropsychiatric traits, and these might therefore warrant further neurobiological investigation. We have made the summary eQTL data available [57] so that others can explore neurodevelopmental antecedents to these and other brain-related conditions.

## Methods

### Samples

Human fetal brain tissue from elective terminations of pregnancy (12–19 PCW) was provided by the Human Developmental Biology Resource (HDBR) (http://www.hdbr.org). Fetal age was determined by the HDBR through foot length and knee-to-heel length measurements. Fetal sex was determined by karyotyping and confirmed in this study through male-specific expression of genes on the Y-chromosome and heterozygosity for X-chromosome markers in females. No other phenotypic or demographic information was available. Brain tissue was obtained frozen and had not been dissected into regions. Total RNA was extracted from half of the available brain tissue from each fetus using Tri-Reagent (Thermo Fisher Scientific), while the other half of each sample was processed for genomic DNA using standard phenol-chloroform extraction.

### Genotyping

Genomic DNA from each sample was genotyped for approximately 710,000 SNPs using the Infinium OmniExpress-24 BeadChip array (Illumina). Genotyping data was subjected to stringent quality control using PLINK v1.9. and the check-bim utility from http://www.well.ox.ac.uk/~wrayner/tools/index.html. Samples with > 5% missing markers, ambiguous sex assignments, or anomalous heterozygosity were excluded. SNPs that were missing in > 5% of samples or had minor allele frequency less than 0.01 were removed, as were A/T and G/C SNPs with minor allele frequencies > 0.4. The SNP strand and ref/alt assignment were updated to match the Human Reference Consortium (HRC) version 1.1, and SNPs where the minor allele frequency differed by > 0.2 from the HRC were removed. Additional genotypes were imputed from the HRC panel using minimac3 and Eagle v2.3 phasing through the Michigan Imputation Server (https://imputationserver.sph.umich.edu/index.html). SNPs were annotated with rsID numbers from dbSNP v149 and converted to GRCh38 coordinates using CrossMap [58] with chain files from the UCSC Genome Browser (https://genome.ucsc.edu/). Non-SNP positions and duplicated sites identified by checkVCF (https://github.com/zhanxw/checkVCF) were filtered out, as were sites with an imputation *r*^2^ less than 0.8, minor allele frequency less than 0.05, or Hardy-Weinberg Equilibrium violation *P* values less than 1 × 10^− 4^. Genotypes from DNA were compared with those from RNA sequencing to confirm matching of genotype and gene expression measures for each sample.

### RNA sequencing library preparation

Total RNA was treated with TURBO DNase (Thermo Fisher Scientific) and purified using the RNeasy Micro kit (Qiagen). Integrity of RNA was assessed using the Agilent 2100 Bioanalyzer. RNA-Seq libraries were prepared using 1 μg of purified total RNA and the TruSeq Stranded Total RNA Library Prep kit (Illumina), depleting ribosomal RNA using the Ribo-Zero Gold reagent (Illumina), and modifying the RNA fragmentation times for lower RIN samples (< 7) according to manufacturer instructions. Library size was assessed using High Sensitivity DNA Analysis kits (Agilent) on the Agilent 2100 Bioanalyzer and libraries quantified using KAPA Library Quantification Kits (KAPA Biosystems) prior to pooling. Libraries were sequenced on the Illumina HiSeq 2500 or HiSeq 4000 systems, generating at least 50 million read pairs (100 million reads) per sample.

### Gene expression analyses

Sequencing adapters were removed from reads with Cutadapt using the Trim Galore! Wrapper script. QC analyses were carried out using FastQC before and after adapter trimming, and additionally using RSeQC [59] after mapping to the GRCh38 human genome reference sequence with HISAT2 [60]. We used bcftools v1.6 to call SNPs in the mapping files and compared them to the imputed genotypes using bcftools gtcheck. Transcript abundance was quantified by pseudoalignment of sequencing reads to transcript sequences derived from the GRCh38 human genome reference sequence and Ensembl (version 81) reference annotation using Kallisto [61]. Reads were aggregated at the gene level using tximport [62] and biomaRt [63]. Between-sample normalization and variance-stabilizing transformation were carried out using DESeq2 [64], and genes/transcripts that had VST-normalized count values > 5 in 10 or more samples were included in all subsequent analyses. The expression matrix was quantile-normalized before and after correcting for covariates (see below), and principal component analysis was used to assess between-sample heterogeneity (Additional file 2: Figure S1). The influence of covariates on gene expression was also quantified using the variancePartition package in R [65] (Additional file 2: Figure S2).

### eQTL discovery

eQTL analyses were carried out using FastQTL [18], using quantile normalized gene expression measures corrected for age, sex, RIN, sequencing batch, the first three genotype principal components (calculated in plink1.9, using LD pruned variants (*r*^2^ > 0.2 in 250 kb windows with a step size of 5 kb)), and 10 hidden confounders estimated through PEER [17]. FDR for each eQTL was calculated by first correcting *P* values for the number of SNPs tested per gene/transcript (within 1 Mb either side of the TSS) through estimation of a beta distribution using a minimum of 1000 permutations (maximum 10,000), and then correcting these *P* values for the number of genes/transcripts tested using Storey’s *q* value method [30]. Details of all steps of these analyses are available at www.github.com/hobrien/GENEX-FB2.

### Comparisons with GTEx adult eQTL data

We obtained multi-tissue meta-analysis results from the Genotype-Tissue Expression (GTEx) Consortium (V7) and matched variants by position and alleles. We determined which of our significant eGenes were assayed by GTEx from the median TPM results, and determine which genes were expressed in each tissue from the normalized expression matrices. Of the 1329 cis-eQTL eGenes (FDR < 0.05) identified in fetal brain, 176 were either not assayed (115 genes) or had expression values below the cut-off in all tissues (61 genes) in the GTEx study. For a further two fetal eGenes, there were no significant *cis-*eQTL that were genotyped by GTEx. For each eGene that was included in at least one GTEx expression matrix, we identified the most significant eQTL variant from our analysis that was assayed by GTEx and extracted the meta-analysis results and *P* values for those eQTLs. For eQTLs that were detected in at least two GTEx tissues, we created a heatmap of *m* values to show the extent of sharing between fetal brain and adult GTEx tissues. Tissue sharing was also quantified by estimating π_1_ (the proportion of true positives [30]) among this set of eQTLs in each GTEx tissue.

### Spatiotemporal expression of fetal eGenes in human brain

We used RNA sequencing data from dissected regions of the human pre- and post-natal brain, available through the BrainSpan project (http://www.brainspan.org/), to explore the regional and developmental expression of the fetal eGenes we identified. FPKM values were log transformed (log_2_[FPKM+ 0.0001]), clustered using Euclidian distances, and heatmaps were made using ggplot2 [66].

### eQTL enrichment analyses

We performed the following enrichment analyses using GARFIELD [38]:

1. Enrichment of eQTL in functional annotation categories
2. Enrichment of mQTL in eQTL
3. Enrichment of variants associated with brain and non-brain complex traits in eQTL

Briefly, GARFIELD tests for enrichment of GWAS associated variants in annotation categories. The user provides the GWAS *P* values for all variants and an annotation file indicating whether variants are located within functional categories to be tested. The software then selects an independent set of variants by sequentially removing variants with *r*^2^ > 0.1 within a 1-Mb window from the most significantly associated variant and then classifies each variant as overlapping a functional category if either the variant or a correlated variant (defined as *r*^2^ > 0.8) is part of the annotation category. Statistical significance is calculated using a generalized linear model, while variants are matched by MAF, distance to the nearest transcription start site, number of LD proxies and CpG and GC content. CpG and GC content were calculated for a region 500 bp up and downstream of the variant using the BSgenome.Hsapiens.UCSC.hg19 R package. All variants from the UK10K were used as the background set of genetic variants. Depending on the particular enrichment analysis, our eQTL data were either considered as the GWAS trait (using the minimum *P* value across all genes/transcripts tested) or the annotation, where all variants associated with at least one gene/transcript at 5% FDR were used to construct the functional category.

#### Enrichment of fetal brain eQTL in functional annotation categories

We tested for significant enrichment of eQTLs in various functional and regulatory features defined using experimentally derived annotations from the ENCODE [8] and Epigenomics Roadmap [9] projects and provided with the GARFIELD software. Enrichment testing was performed for fetal brain transcript eQTLs with *P* values equivalent to FDR < 0.05. We tested the default of all features in all assayed cells/tissues (1004 tests), defining *P* values below the Bonferroni-corrected threshold of *P* < 4.98 × 10^− 5^ as significant.

#### Enrichment of fetal brain mQTLs in eQTL

To test for enrichment of genetic variants associated with DNA methylation in the developing brain within fetal brain eQTL, we used a DNA methylation QTL (mQTL) dataset that we published previously using an overlapping set of fetal brain samples [10]. All FDR < 0.05 eQTLs were used to define an annotation category. Enrichment was tested using the set of mQTL that we previously identified at a Bonferroni-corrected threshold *P* < 3.69 × 10^− 13^.

#### Enrichment of variants associated with brain and non-brain complex traits in eQTLs

GWAS results for attention deficit hyperactivity disorder [31], anorexia nervosa [32], autism spectrum disorder [33], bipolar disorder [34], major depressive disorder [35], neuroticism [26], schizophrenia [36], body mass index [39], coronary artery disease [40], inflammatory bowel disease [41], height [42], and type 2 diabetes [43] were downloaded, and the *P* values for all variants were reformatted as input to GARFIELD. All 5% FDR eQTLs for eTranscripts were used to define an annotation category. We tested for enrichment in fetal brain eQTLs of variants associated with each trait at multiple significance thresholds (*P* < 5 × 10^− 5^, 5 × 10^− 6^, 5 × 10^− 7^, 5 × 10^− 8^). In total, we tested 12 traits and 4 significance thresholds, highlighting enrichments that surpassed the Bonferroni corrected threshold *P* < 0.001.

### Testing for pleiotropic association with neuropsychiatric traits

We tested for joint associations between fetal brain eQTL and GWAS signals using summary-statistics-based Mendelian randomization (SMR) [44]. We tested all eGenes/eTranscripts with eQTLs detected at FDR < 0.05, using linkage disequilibrium information from the samples used for our eQTL analysis. GWAS minor allele frequencies were obtained from the 1000 Genomes Project (European populations). SMR *P* values were Bonferroni corrected for the number of eGenes/eTranscripts tested. The HEIDI test was used to screen out joint associations that are likely to be driven by linkage using a nominal *P* value threshold of 0.05.

We additionally investigated potential sharing of eQTLs identified by the CommonMind Consortium in the adult human brain [49] that have been implicated in schizophrenia or neuroticism in the SMR study of Hauberg and colleagues [45]. For this, we restricted our analyses to the fetal brain eGenes detected at FDR < 0.05 that overlapped with those implicated by Hauberg et al. in neuropsychiatric traits, and explored potential linkage disequilibrium between the top adult brain SMR eQTL and the most significant fetal brain eQTL for each gene. We report instances where the top adult brain SMR eQTL and the top eQTL for that gene in fetal brain have an *r*^2^ > 0.8 in our samples, suggesting shared causal variants.

## Additional files

Additional file 1: **Tables S1**-**S10.** containing sample data, top eQTL for all significant eGenes and eTranscripts (FDR < 0.05), results of enrichment analyses and results of SMR analyses in relation to neuropsychiatric traits. (XLSX 987 kb)

Additional file 2: **Figures S1**-**S8.** All supplementary figures. (PDF 1212 kb)
