# Supplementary material for: Expression quantitative trait loci in the developing human brain and their enrichment in neuropsychiatric disorders
Source: Genome Biol. 2018 Nov 12;19:194. doi: 10.1186/s13059-018-1567-1 (PMC6231252; doi:10.1186/s13059-018-1567-1)
Supplement: Supplementary file 2 — Figures S1-S8. All supplementary figures. (PDF 1212 kb) [file 13059_2018_1567_MOESM2_ESM.pdf]

Supplementary Figures

Expression quantitative trait loci in the developing human brain and their enrichment in neuropsychiatric disorders

Heath E. O'Brien [1], Eilis Hannon [2], Matthew J. Hill [1], Carolina C. Toste [1], Matthew J. Robertson [1], Joanne E. Morgan [1], Gemma McLaughlin [3], Cathryn M. Lewis [3], Leonard C. Schalkwyk [4], Lynsey S. Hall [1], Antonio F. Pardiñas [1], Michael J. Owen [1], Michael C. O'Donovan [1], Jonathan Mill [2], Nicholas J. Bray [1]

[1] MRC Centre for Neuropsychiatric Genetics & Genomics, Division of Psychological Medicine & Clinical Neurosciences, Cardiff University, Cardiff, United Kingdom

[2] University of Exeter Medical School, University of Exeter, United Kingdom

[3] Institute of Psychiatry, Psychology & Neuroscience, King's College London, London, United Kingdom

[4] School of Biological Sciences, University of Essex, Colchester, United Kingdom

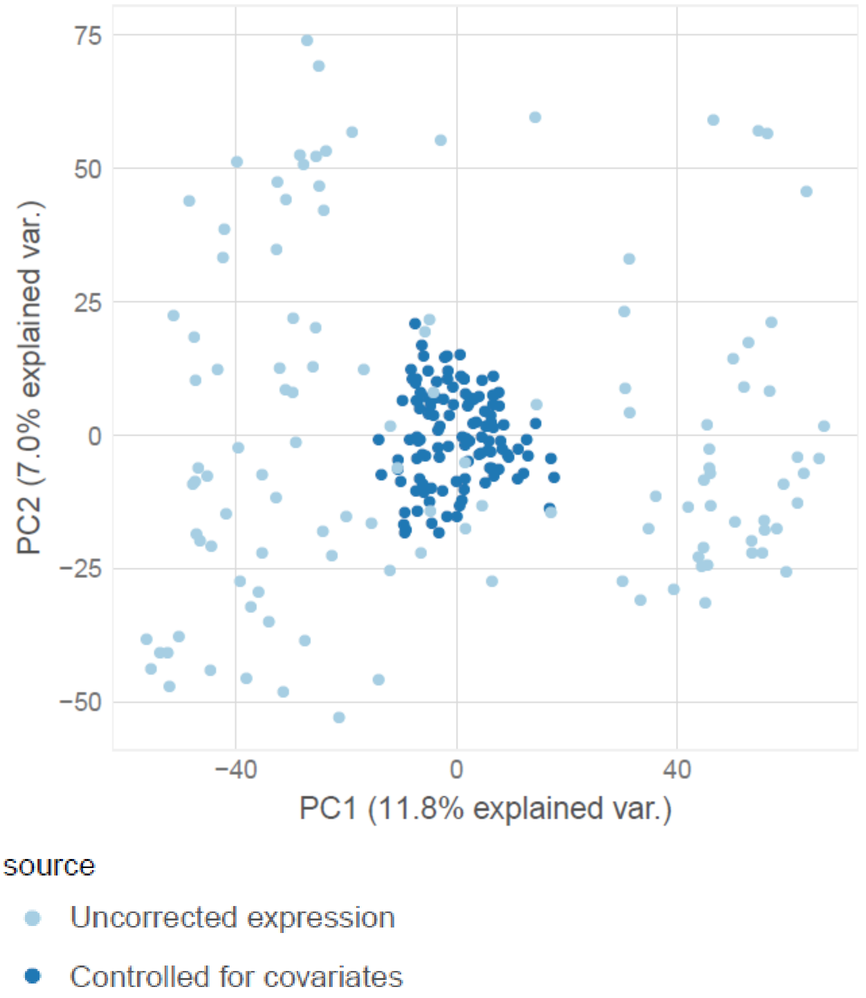

Figure S1. Principal component analysis of normalized, VST transformed expression data before and after controlling for covariates and PEER factors (Stegle et al, 2012). Expression matrices were quantile-normalized before combining.

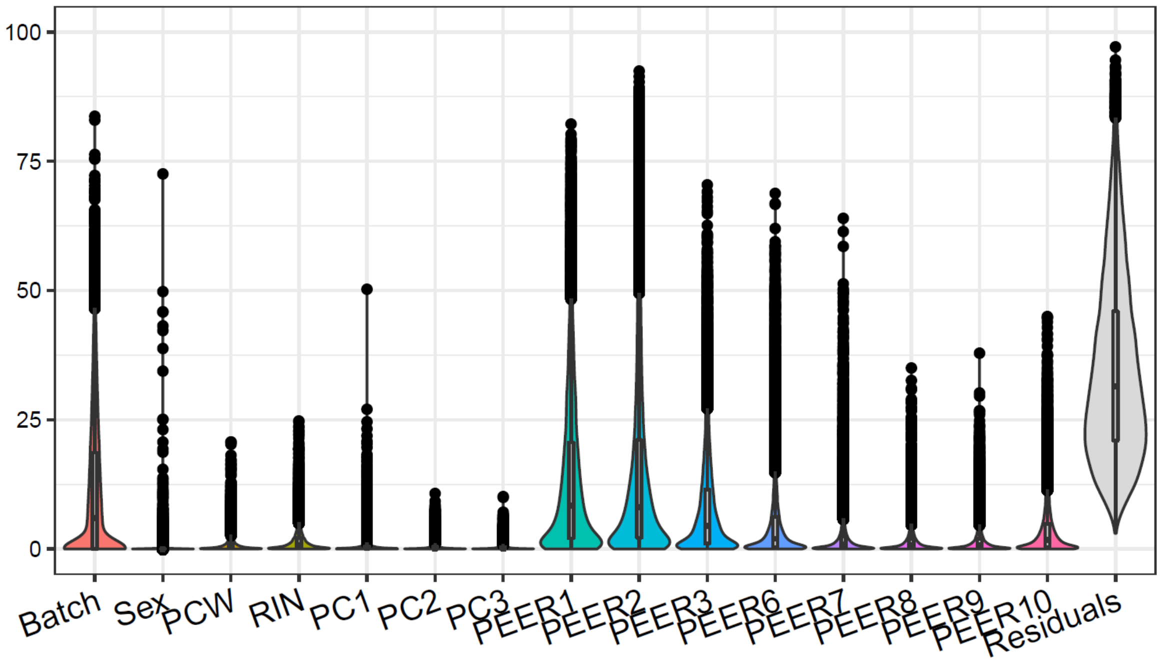

Figure S2. Contribution of covariates and PEER factors to expression variance. Variance was partitioned for each gene using linear models fit with the variancePartition (Hoffman et al, 2016) package in R. PEER factors 4 and 5 were excluded because of their high correlation to other covariates. The residuals, which were used for the eQTL analysis, account for 20% or more of the variance in 75% of genes.

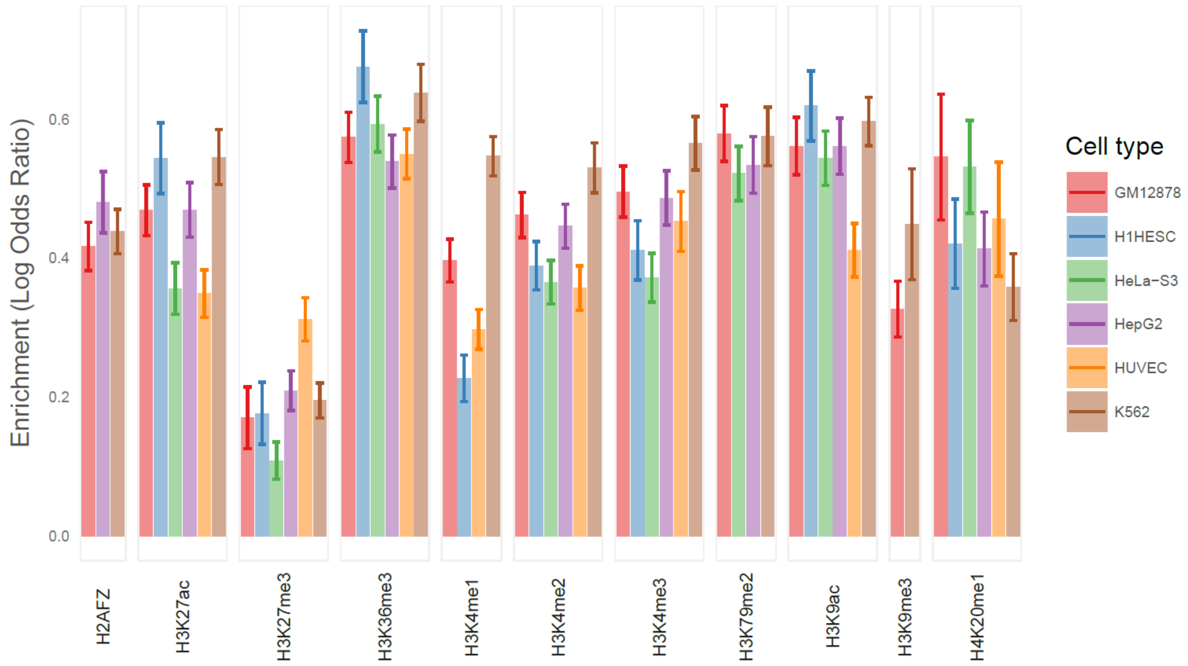

Figure S3: Enrichment of fetal brain transcript eQTL within genomic regions marked by histone modifications indicative of regulatory activity. Histone modifications were identified by the ENCODE and Roadmap Epigenomics Consortium projects in 6 human cell lines. Enrichments are expressed as the natural log of odds ratios, with error bars representing 95% confidence intervals. GM12878 = lymphoblastoid cell line; H1HESC = embryonic stem cell line; HeLa-S3 = cervix adenocarcinoma cell line; HepG2 liver carcinoma cell line; HUVEC = umbilical vein

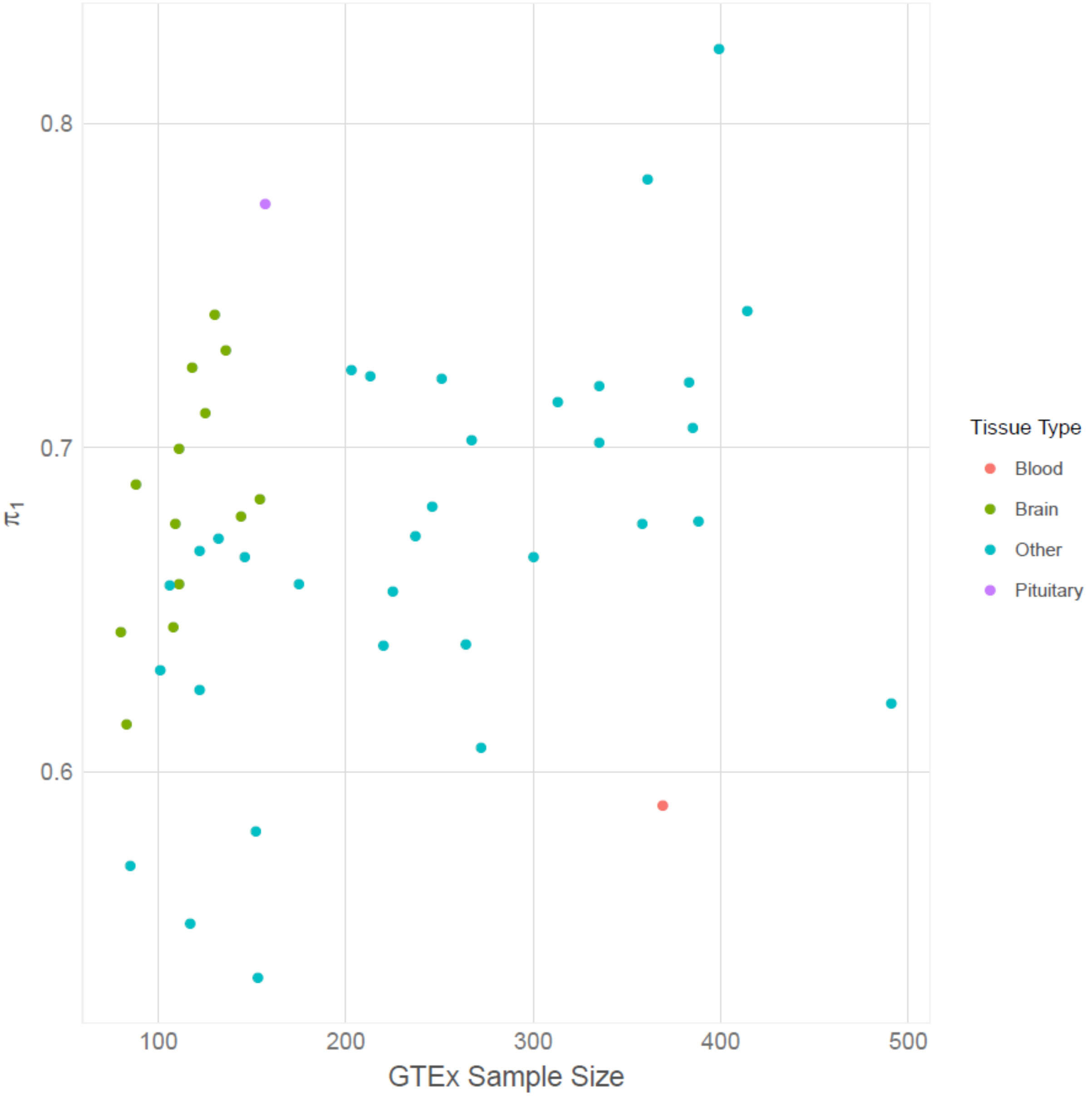

Figure S4: Estimated proportions of top fetal brain eQTLs that are true positive eQTLs (π1; Storey et al, 2003) in adult GTEx tissues versus GTEx sample size. Only genes that were analysed by GTEx were included in calculations. In general, higher π1 is observed with larger GTEx tissue sample sizes, but adult brain regions have relatively high replication rates despite small samples sizes while replication in blood is relatively low despite a larger GTEx sample size.

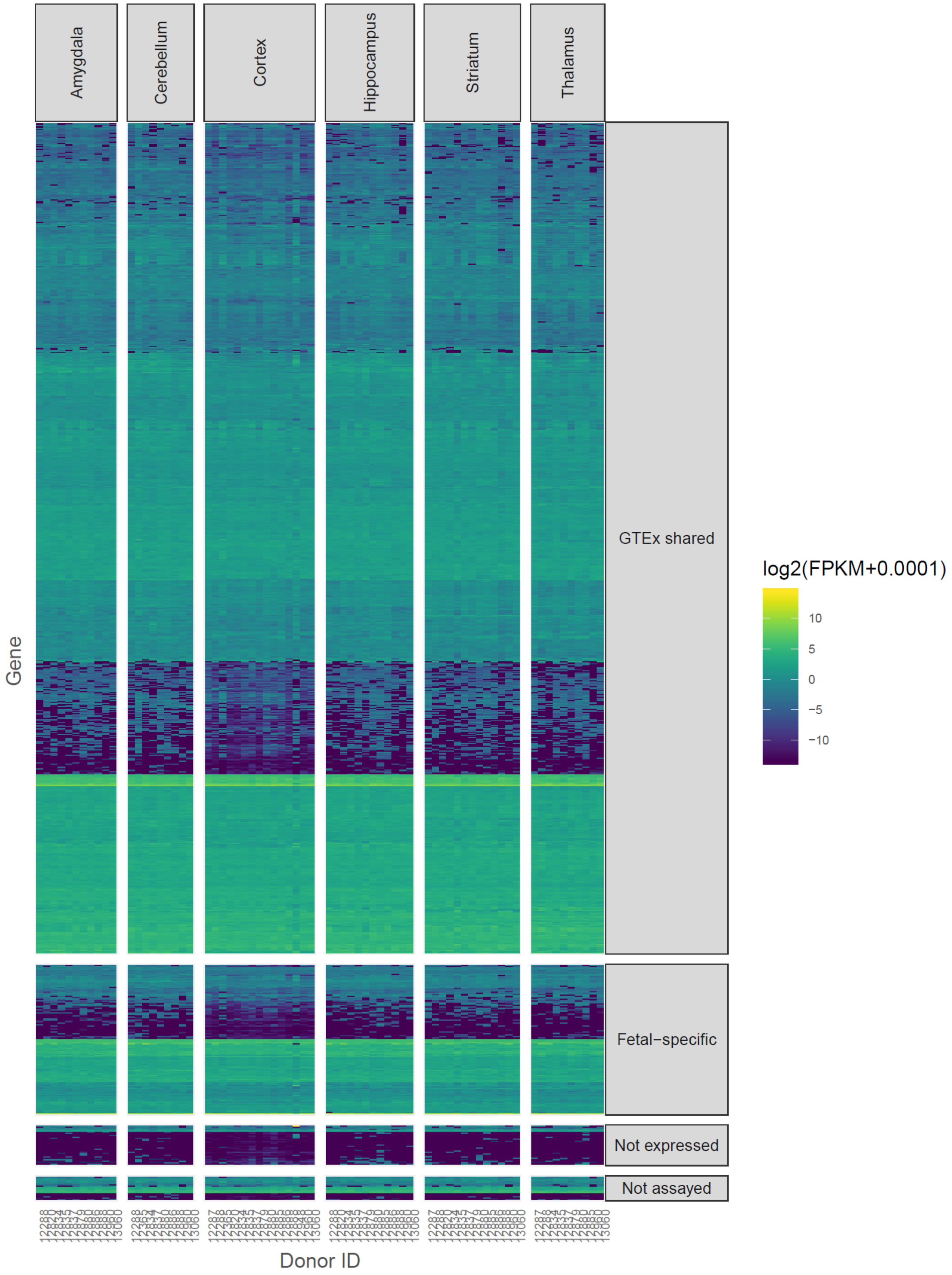

Figure S5: Heatmap showing expression of fetal eGenes across regions of the developing human brain (second trimester). Expression data are from the BrainSpan Developmental Transcriptome

(http://www.brainspan.org/). Cortex: mean of dorsolateral prefrontal cortex, ventrolateral prefrontal cortex, primary auditory cortex (core), primary visual cortex (striate cortex, area V1/17), anterior (rostral) cingulate (medial prefrontal) cortex, primary motor-sensory cortex, primary somatosensory cortex (area S1, areas 3,1,2), primary motor cortex (area M1, area 4), posterior (caudal) superior temporal cortex (area 22c), inferolateral temporal cortex (area TEv, area 20), posteroventral (inferior) parietal cortex and orbital frontal cortex. Thalamus: mean of mediodorsal nucleus of thalamus and dorsal thalamus. Cerebellum: mean of cerebellum and cerebellar cortex. Genes are divided into those where eQTL are predicted to be shared with adult GTEx tissues, those with putative fetal-specific eQTL, those that had expression values below the cut-off in all adult GTEx tissues and those that were not assayed by GTEx.

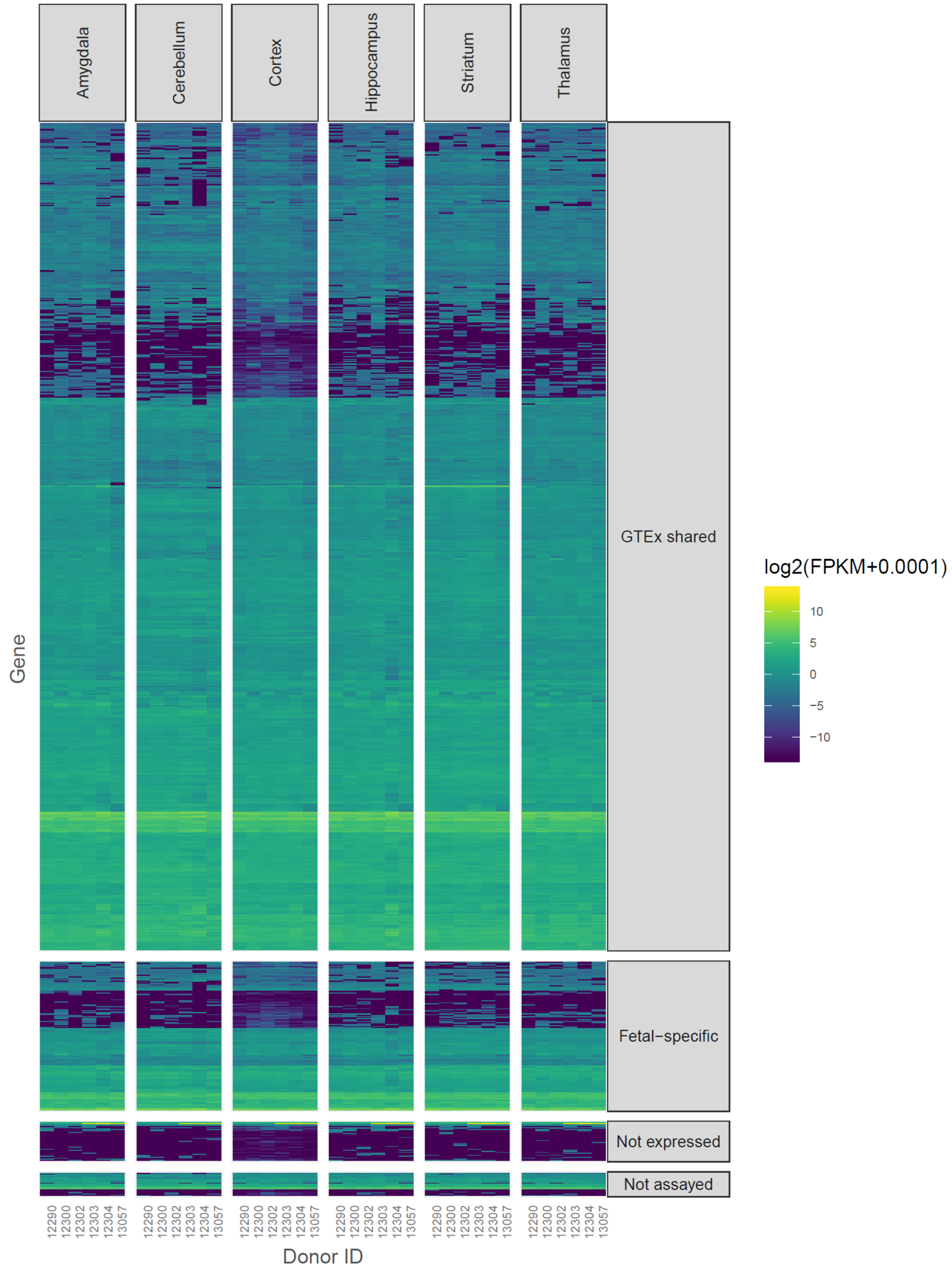

Figure S6: Heatmap showing expression of fetal eGenes across regions of the adult human brain.

Expression data are from the BrainSpan Developmental Transcriptome (http://www.brainspan.org/).

See Figure S5 legend for explanation of brain regions and gene classification in relation to GTEx data.

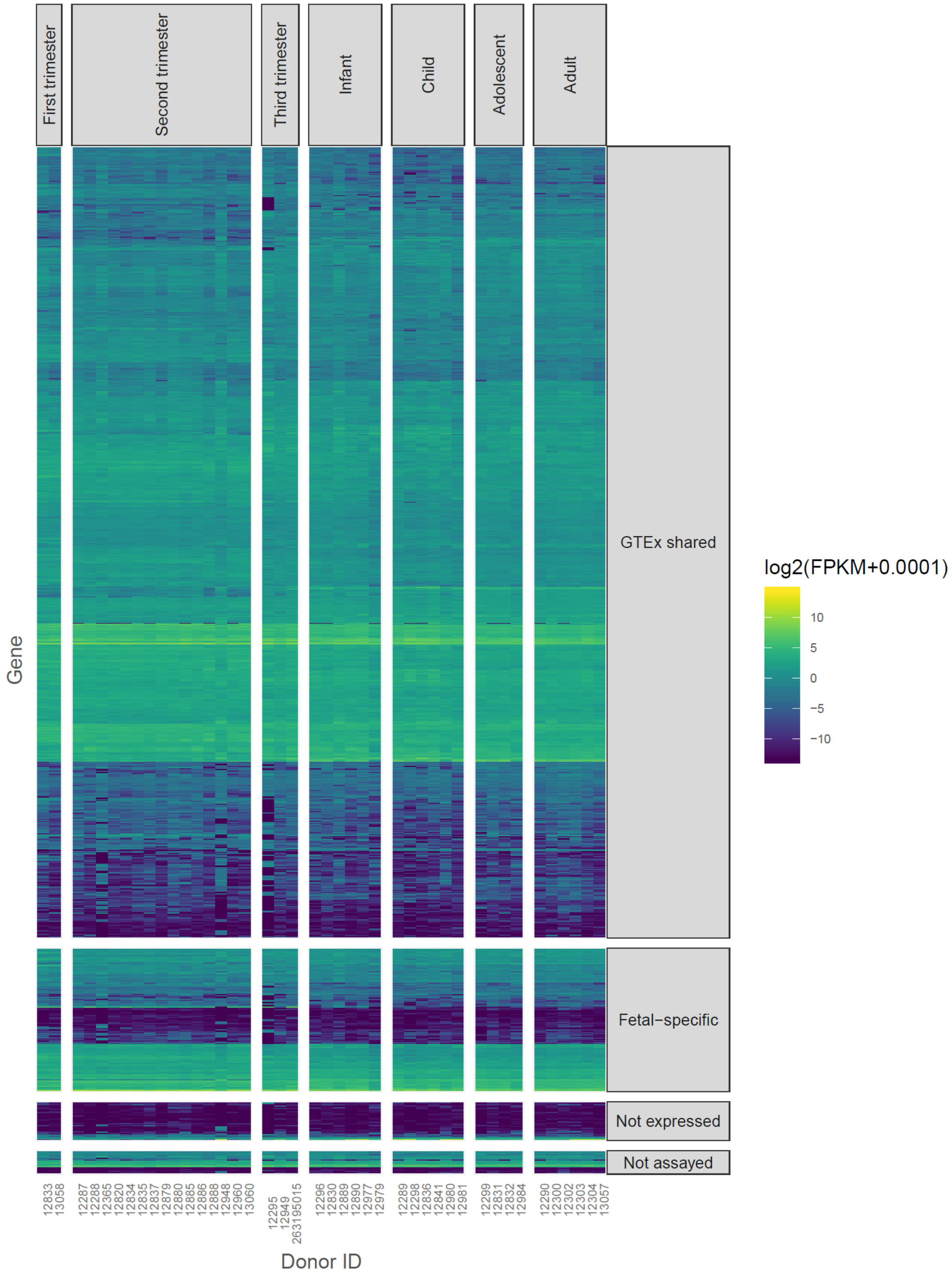
First trimester
Second trimester
Third trimester
Infant
Child
Adolescent
Adult
GTEx shared
Fetal−specific
Not expressed
Not assayed
Gene
log2(FPKM+0.0001)
10
5
0
−5
−10
12833
13058
12287
12288
12365
12820
12834
12835
12837
12879
12880
12885
12886
12888
12948
12960
13060
12295
12949
263195015
12296
12830
12889
12890
12977
12979
12289
12298
12836
12841
12980
12981
12299
12831
12832
12984
12290
12300
12302
12303
12304
13057
Donor ID

Figure S7: Heatmap showing expression of fetal eGenes in the human cerebral cortex across developmental stages. Expression data are from the BrainSpan Developmental Transcriptome (http://www.brainspan.org/). First trimester: 8-9 post-conception weeks. Second trimester: 12-25 post-conception weeks. Third trimester: 26-37 post-conception weeks. Infant: 0-2 years. Child: 3-11 yrs. Adolescent: 13- 19 yrs. Adult: 21 yrs and older. See Figure S5 legend for explanation of gene classification in relation to GTEx data.

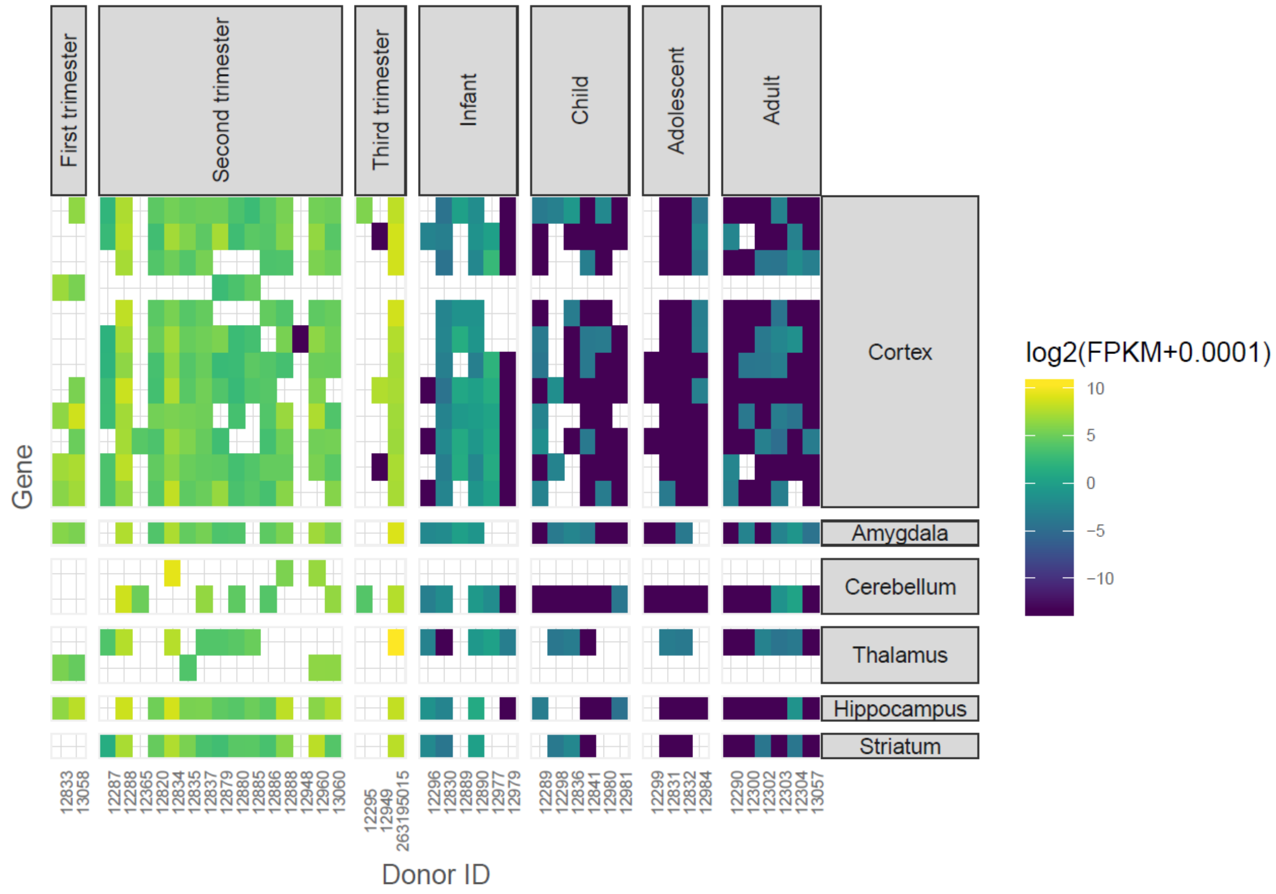

Figure S8: Heatmap showing expression of the fetal-specific eGene *HBG1* across human brain regions and developmental stages. Expression data are from the BrainSpan Developmental Transcriptome (http://www.brainspan.org/). First trimester: 8-9 post-conception weeks. Second trimester: 12-25 post-conception weeks. Third trimester: 26-37 post-conception weeks. Infant: 0-2 years. Child: 3-11 yrs. Adolescent: 13- 19 yrs. Adult: 21 yrs and older. See Figure S5 legend for explanation of brain regions
